# Cell Cycle Plasticity and Heterogeneity: An Underappreciated Feature of Cancer and Treatment Response

**DOI:** 10.47248/chp2502030015

**Published:** 2025-09-25

**Authors:** Erik S. Knudsen, Thomas N. O’Connor, Agnieszka K. Witkiewicz

**Affiliations:** Department of Molecular and Cellular Biology, Roswell Park Comprehensive Cancer Center, Elm and Carlton Street, Buffalo, NY 14263, USA;

**Keywords:** Cell cycle, cancer, plasticity, cyclin, CDK, CDK inhibitor, palbociclib

## Abstract

Progression through the mammalian cell cycle is a highly regulated process to maintain tissue homeostasis. The key regulators of cell cycle transitions are cyclin-dependent kinase (CDK)/Cyclin complexes that phosphorylate substrates such as the RB tumor suppressor to facilitate cellular division. The regulation of G1/S is of particular significance in cancer and is affected by numerous tumor suppressors and oncogenes. Historically, the cell cycle was viewed as a rigidly regulated process, but recent evidence has revealed significant flexibility and differential CDK/Cyclin dependencies across tumor types. These heterogeneous features of cell cycle control have implications for the etiology of different tumor types as well as the response to multiple therapeutic modalities. Most notably, adaptive responses in cell cycle regulatory circuits can contribute to acquired resistance in a variety of contexts, underscoring the importance for tumor biology and disease treatment.

## 1. Overview of G1/S Control

**Figure 1:**
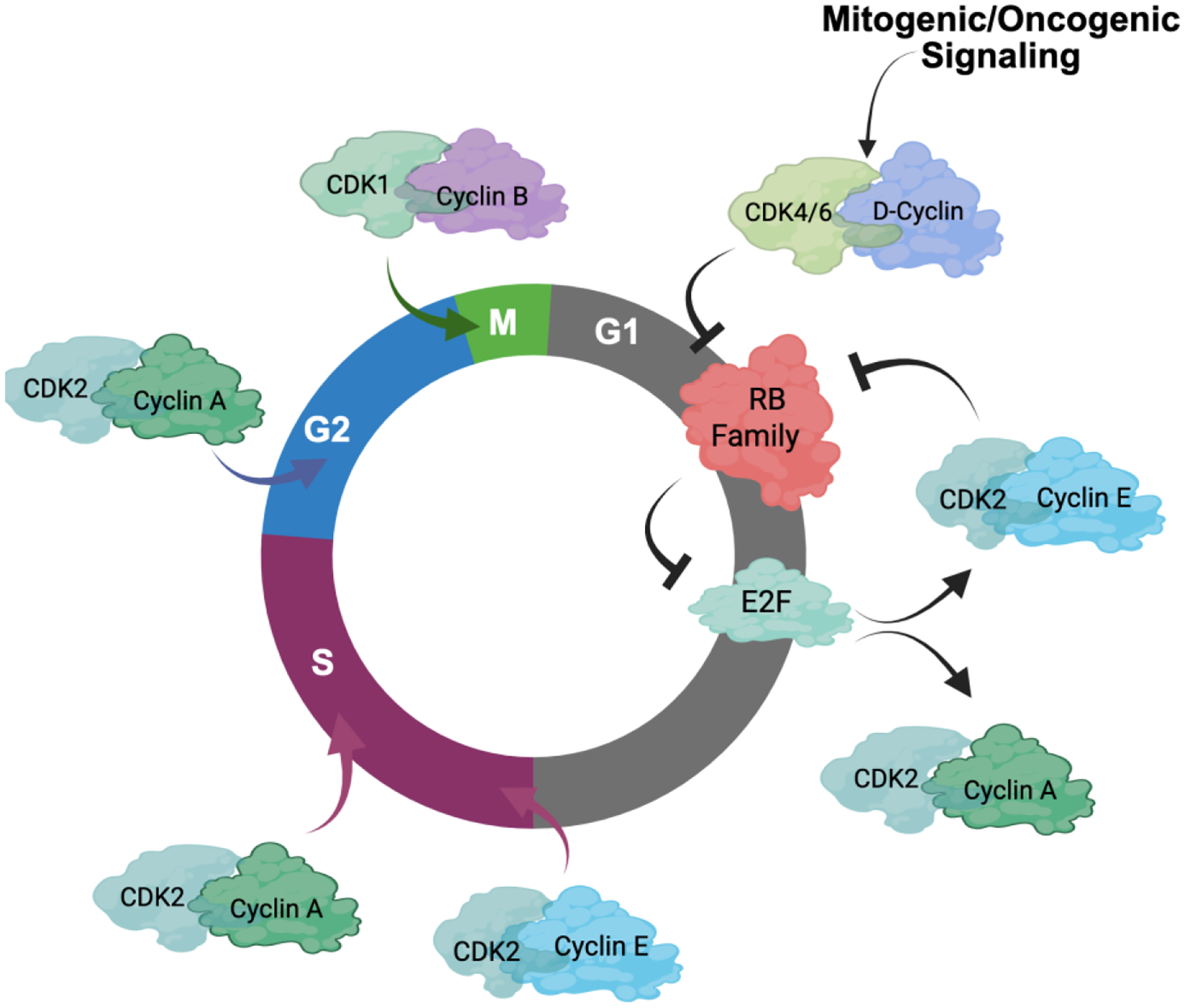
Conventional schematic of the cell cycle. Mitogenic or oncogenic signals impinge on the cell cycle at the level of CDK4/6 complexes. These initiate the phosphorylation of RB and related proteins to release E2F transcription factors and mediate the activation of CDK2. CDK2 contributes to the phosphorylation of RB-family proteins and drives subsequent cell cycle transitions. Subsequent CDK1/Cyclin B complex activation promotes entry into mitosis. Figure adapted from Knudsen *et al*., 2025 [13] in compliance with the article’s Creative Commons license.

**Figure 2:**
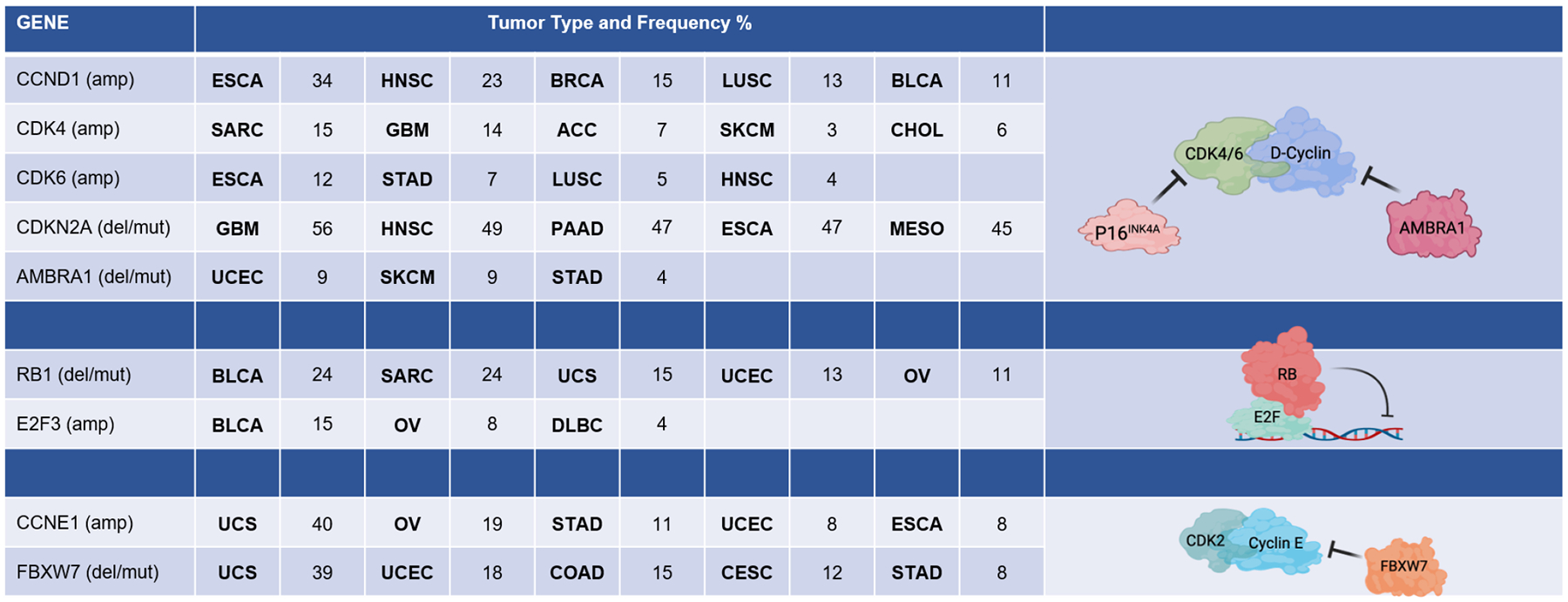
Cell cycle genes frequently altered in various cancers. The table summarizes data from The Cancer Genome Atlas (TCGA) pan-cancer atlas queried through cBioportal. ESCA, esophageal cancer; HNSC, head and neck squamous cell carcinoma; BRCA, breast cancer; LUSC, lung squamous cell carcinoma; BLCA, bladder cancer; SARC, sarcoma; GBM, glioblastoma; ACC, adenoid cystic carcinoma; SKCM, skin cutaneous melanoma; CHOL, cholangiocarcinoma; ESCA, esophageal cancer; STAD, stomach adenocarcinoma; PAAD, pancreatic adenocarcinoma; MESO, mesothelioma; UCEC, uterine corpus endometrial carcinoma; UCS, uterine carcinosarcoma; OV, ovarian cancer; DLBC, diffuse large B-cell lymphoma; COAD, colon adenocarcinoma; CESC, cervical squamous cell carcinoma.

The key regulatory step controlled by oncogenic networks is traversal of the G1/S phase transition of the cell cycle [1]. Oncogenes such as *KRAS*, *ERBB2*, and *EGFR* highjack conventional mitogenic networks to promote cell cycle progression. Mitogens are believed to interface with the cell cycle through cyclin-dependent kinase (CDK) 4/6 and D-type cyclin complexes (Figure 1) [1–4]. Growth factors induce the expression of D-type cyclins (Cyclin D1, D2, or D3) in a variety of tissues and promote the stabilization and activation of CDK4/6 kinases through multiple mechanisms [3]. The importance of CDK4/6 in tumor development is evidenced by the frequent amplification or aberration of *CDK4*, *CDK6*, and *CCND1* encoding Cyclin D1 in many cancers (Figure 2) [5–7]. Conversely, negative regulators of Cyclin D1 or CDK4/6 are tumor suppressors. For example, *CDKN2A*, which encodes the tumor suppressor p16INK4A, is a negative regulator of CDK4/6 and is frequently lost in many cancers (Figure 2) [8–10]. The AMBRA1 tumor suppressor mediates the degradation of Cyclin D1 [11,12] (Figure 2). Thus, it has been presumed that just as *KRAS* mutations bypass dependence on mitogenic signals, alterations in the cell cycle bypass upstream processes that enable unregulated proliferation.

A key substrate of CDK4 and CDK6 activity is the retinoblastoma tumor suppressor (RB) [4,14,15]. RB was the first tumor suppressor identified and, along with its related pocket protein family members p107 and p130, conventionally serves to coordinate gene expression programs controlled by the E2F transcription factor (Figure 1 and 2) [16–18]. The E2F transcriptional program encodes many “pan-essential” genes that promote DNA replication (e.g., *MCM7*, *CDC6*, *CDT1*) and mitotic progression (e.g., *PLK1*, *AURKA*, *CCNB1*) [17,19–22]. In its active, hypophosphorylated state, RB binds E2F and serves as a potent transcriptional co-repressor [23,24]. Phosphorylation of RB, as well as p107 and p130, by CDKs releases RB from E2F and alleviates this transcriptional repression, enabling progression through G1/S [17,25]. A number of E2F genes are amplified in cancers including E2F3 and E2F1 [26,27]. Many targeted therapies act to prevent RB phosphorylation and, thus, repress this central transcriptional circuit. As would be expected, this RB/E2F transcriptional circuit is commonly deregulated in cancer and is generally associated with cancers that have poor prognosis [28,29].

Another driver of the G1/S transition is Cyclin E, encoded by *CCNE1* [30–32]. Like *CCND1*, *CCNE1* amplification occurs in several cancer types (Figure 2). Cyclin E interacts with CDK2 and is believed to play an important role in driving G1/S progression as well as coordinating initiation of DNA replication and transit through G2 phase of the cell cycle [32,33] (Figure 1). The regulation of Cyclin E is complex and involves transcriptional and post-translational modifications that modulate protein accumulation. Some of these processes are related to oncogenesis, as the tumor suppressor FBXW7 modulates Cyclin E stability as one of its targets [34]. Cyclin E is considered an E2F-regulated gene and has been proposed to function at least in part downstream of RB [35,36]. However, Cyclin E and CDK2 activity is also mitogen regulated, albeit the mechanisms are not as clearly understood as the mitogen regulation of CDK4/6. Yet, one notable mechanism is the expression of p21Cip1 and p27Kip1, which are endogenous CDK2 inhibitors, that are induced by a variety of signaling events and serve to constrain CDK2 activity [37–39].

## 2. Cell Cycle Definitions

With thousands of review articles published showing a common cell cycle regulatory structure, terms like plasticity and heterogeneity may seem out of place. The term *plasticity* is defined as the quality of being easily shaped or molded. In a biological sense, this would include alterations in a particular process or mechanism either occurring stochastically or under particular stressors. In one sense, plasticity and evolution are related, with a definition of *evolution* being the gradual development of something, especially from a simple to a more complex form. Perhaps the time scale is different but conceptually both share similarities in changes occurring from an initial primordial state. From a cell cycle perspective, such plasticity or evolution could be from using CDK4 to initiate the cell cycle to using CDK2, or perhaps the evolution to an RB-deficient state under selective pressure. Data from mouse models with CDK or Cyclin deletions indicate that such transitions can occur under the pressure of organismal survival. *Heterogeneity* in contrast reflects an end state; the quality or state of being diverse in character or content. For example, many solid cancers have heterogeneity at diagnosis in terms of predominant CDK/Cyclin or RB status associated with a given cancer. As indicated above, this heterogeneity can be related to the genetics of the tumor, such as amplifications and epigenetic features. These definitions are important as plasticity and evolution ultimately promote heterogeneity and these processes are continual during cancer etiology, tumor progression, and acquired resistance to therapy. In addition to states that promote tumor cell proliferation, as is the focus here, there are clearly other adaptations such as tumor dormancy [40], polyploid giant cells [41], whole-genome doubling [42], and other cell cycle processes that may ultimately lead to increased fitness as a result of alterations in cell cycle control.

## 3. Heterogeneous Nature of Cancer Cell Cycles

While the cell cycle is often drawn, by us and others, as a circle with different CDK and cyclin activities promoting various transitions (e.g., Figure 1) [43,44], this summation belies the intrinsic complexity of cell cycle and redundancies in most facets of G1/S control [45,46]. Based on biochemical activities, it would be expected that many cell cycle drivers such as CDK4 or CDK2 would be pan-essential. However, mice can develop normally, for the most part, in the absence of CDK2 and develop with tissue-selective deficits upon CDK4 or CDK6 deletion [47,48]. While the combination of CDK4 and CDK6 deletion is lethal in mice, it has a limited impact on the development of multiple cell lineages, with these cells successfully progressing through the cycle [49]. In fact, it is possible to ablate the “interphase” CDKs (CDK4, CDK6, and CDK2) to yield a cell cycle that is driven by CDK1 in mice [50]. In the context of tumor models such plasticity is also apparent where Cyclin D3 can compensate for Cyclin D1 deletion in ERBB2 mammary tumorigenesis [51]. Thus, there is significant redundancy relative to individual CDK genes and an inherent ability to adapt and use “non-canonical” CDK/Cyclin complexes to facilitate G1/S transition.

Large-scale CRISPR screens in cancer cells have further expanded our understanding of key CDK and cyclin gene dependencies [52,53]. These data suggest that the importance of a given CDK or cyclin is associated with a complex interaction between cancer genetics, epigenetic states, and features that likely pertain to tumor lineage. For example, dependence on *CDK4* is associated with amplification of *CCND1* or expression of the Cyclin D1 protein [52,53]. In contrast, dependence on *CDK2* is associated with high expression of Cyclin E and the expression of p16INK4A, limiting the ability of these cells to use CDK4/6 for cell cycle progression [54–57]. In this context, cell lineage is also an important feature related to cell cycle control. For example, multiple hematological cancers preferentially utilize CDK6 for proliferation, which is consistent with CDK6 use in normal hematopoietic progenitor proliferation as well [49,58,59]. Further, advancement of single-cell analyses has provided the opportunity to track these dependencies at higher resolution over time to help define the alterations of cell cycle drivers in a given tumor [60–65]. For example, single cell analyses can illustrate rapid adaptation to CDK4/6 inhibition which can occur through CDK2/Cyclin D1 complexes [66] and yield enhanced sensitivity to CDK2 inhibition [62].

**Figure 3:**
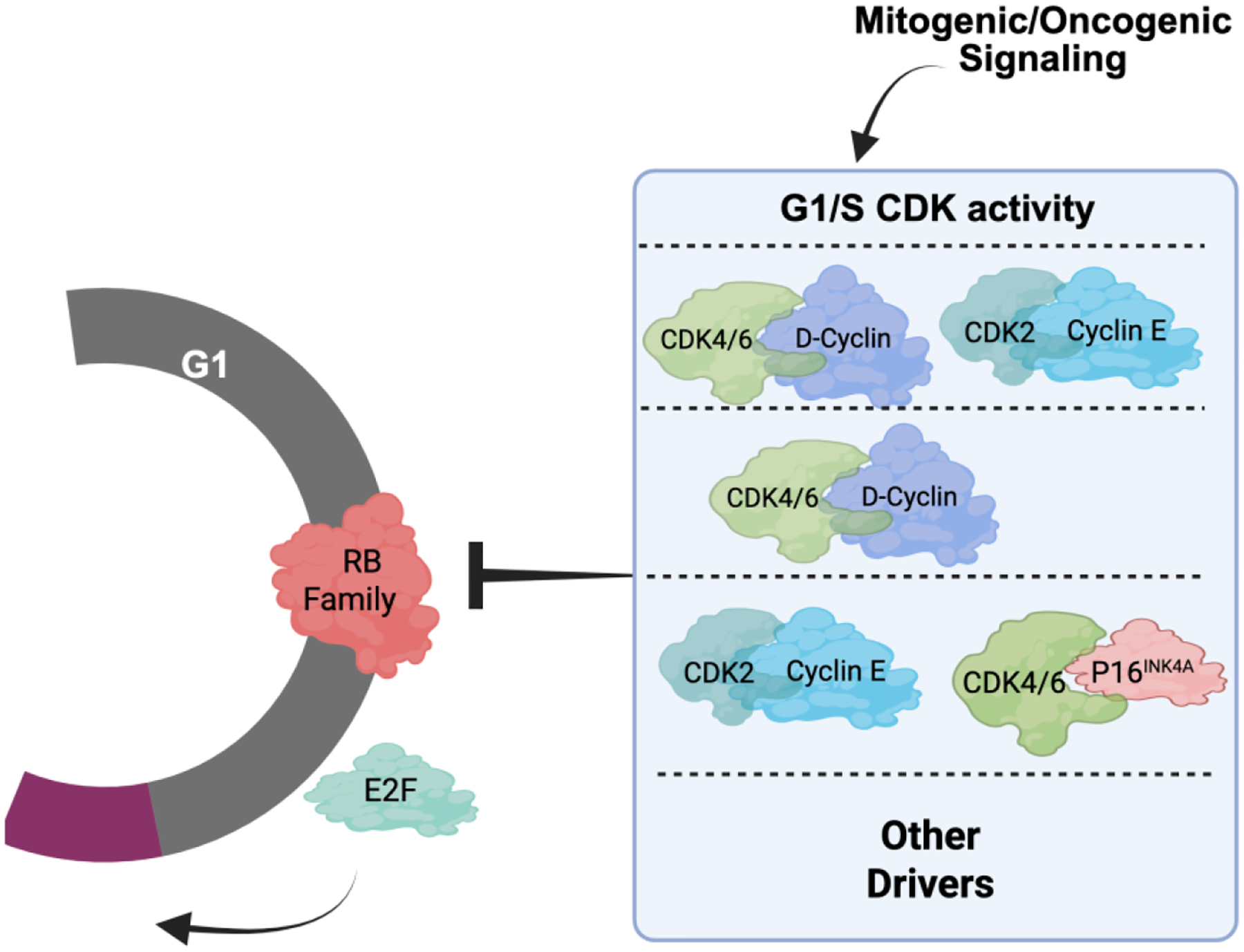
Alternative schematic of G1/S cell cycle control. A host of different G1/S CDK activities can yield transit through G1/S. It is possible that a variety of drivers beyond the conventional CDK4/6 and CDK2 complexes can promote cell cycle progression. Figure adapted from Knudsen *et al*., 2025 [13] in compliance with the article’s Creative Commons license.

Taking these data into consideration, there is perhaps no singular canonical cancer cell cycle, but rather different tumors have a preferred cell cycle for the G1/S transition (Figure 3). This concept of varying cell cycle states allows individual tumors to have distinct cell cycles that are shaped by multiple features of that specific tumor. By interrogating many tumors, it is possible to start to infer common paths that may be relevant for tumor etiology or therapeutic responses. For example, many ovarian tumors express high levels of Cyclin E and p16INK4A with relatively low levels of Cyclin D1 [54–57]. This configuration would predict more dependence on CDK2. In contrast, most hormone receptor-positive, human epidermal growth factor receptor 2-negative (HR+/HER2-) breast cancer presentations have high expression of D-type cyclins (*i.e*., Cyclin D1), with low Cyclin E and p16INK4A that could be associated with sensitivity to CDK4/6 inhibitors that are FDA approved for that tumor type [46,52,67]. While these heterogeneous cell cycle states can pre-exist as indicated above, they can adapt to various stresses including therapies and thus are not static.

## 4. Cell Cycle Deregulation in Cancer

**Figure 4:**
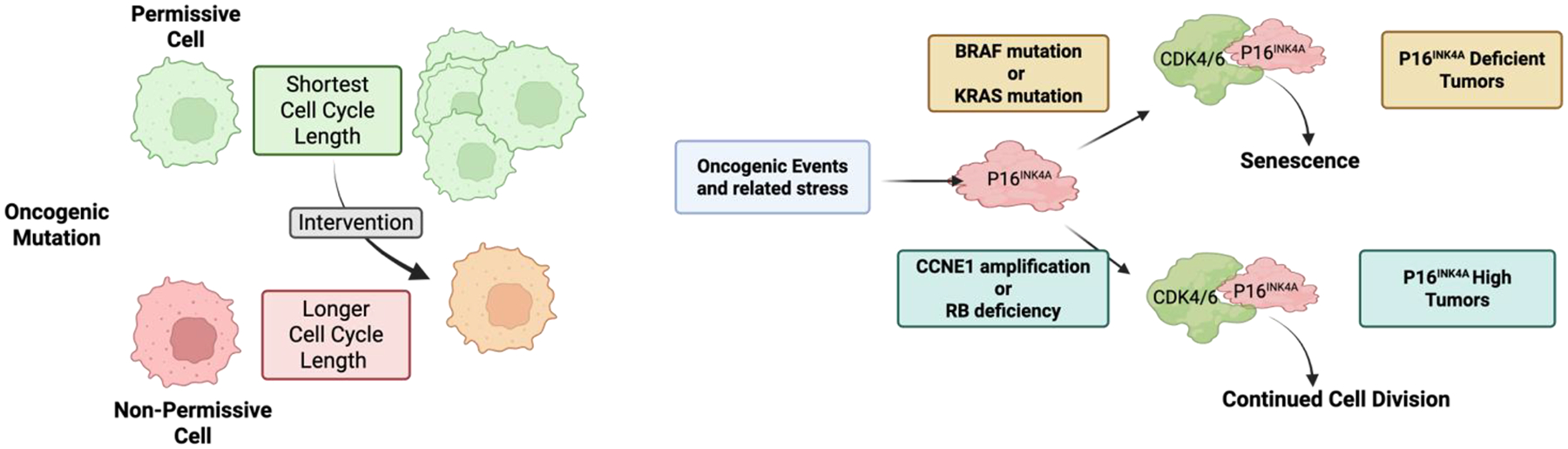
Distinct factors that control tumor development. (**A**) It is well-established that oncogenic mutations can drive tumor development in only a subset of permissive cells. It has recently been shown that those cells where the oncogenic event elicits the shortest cell cycle length is associated with the permissive state. A number of interventions that lengthen the cell cycle transit time were shown to suppress tumor development. (**B**) Oncogenic events represent an intrinsic cellular stress that can lead to the upregulation of the endogenous CDK4/6 inhibitor p16INK4A. This event can drive senescence and p16INK4A must be downregulated for tumor development, as is seen in most tumors driven through the KRAS-BRAF signaling pathway. In contrast, CCNE1 amplification or RB deficiency yield a cell cycle that does not require CDK4/6. Thus, the tumors retain p16INK4A.

As noted above, several key mediators of G1/S control are oncogenes and tumor suppressors. Since many of these genes are in fact multi-functional, there remains controversy related to whether cell cycle control and proliferation or other processes are key in driving tumor development. For example, while Cyclin E overexpression can advance cell cycle transitions, Cyclin E can exert replication stress and features of chromosome instability that could contribute to cancer etiology [32,68]. Similarly, while RB deficiency deregulates cell cycle, RB loss has broad effects related to lineage states and epigenetic programs beyond cell cycle [18,44,69]. Such complexities are perhaps part of the reason why most cell cycle regulators have a limited spectrum of relevance in disease etiology. For example, if RB is a global mediator of cell cycle control, then why is germline RB loss associated primarily with pediatric retinoblastoma, with secondary malignancies (e.g., soft-tissue sarcoma and osteosarcoma) emerging sporadically and much later in life [70]? Similarly, germline CDKN2A deficiency (while in the same pathway as RB) is associated with familial atypical multiple mole melanoma syndrome that yields increased risk for specifically melanoma and pancreatic cancer [71]. A new study interrogating the cell of origin for RB1 deficiency showed that a key determinant of tissue tropism is the ability of select cells to deregulate cell cycle transit [72], defining the cells of origin as having the shortest cell cycle length (Figure 4A). By a variety of genetic permutations/interventions, it is possible to restrict tumor development by lengthening cell cycle transit [72]. This study employed multiple oncogenic mutations beyond RB1 loss and indicates that the tumor spectrum is highly related to those cells that are most susceptible to a given perturbation changing cell cycle transit times to fuel tumor development.

Outside of direct permutations to the cell cycle machinery, veritably all cancers are characterized by classic cancer hallmarks such as mitogen-independent growth and escape from proliferative limitations, among others [73]. In the context of many oncogenic drivers, there are specific mechanisms through which they promote deregulation of cell cycle (e.g., upregulation of Cyclin D1 and depletion of p27Kip1) [74,75]. Oncogenes in the RAS-BRAF-MAPK pathway can potently drive proliferation but also have the capacity to drive oncogene-induced senescence [76,77] (Figure 4B). This particular response to oncogenic signaling is caused at least in part by the upregulation of p16INK4A, which inhibits CDK4/6 to limit cell cycle progression [77,78]. This phenomenon is likely very effective in limiting tumor development, as benign nevi that are driven by mutant BRAF are arrested in G1 by p16INK4A and can remain in that non-malignant state [79]. Presumably, this is a relatively general mechanism to suppress oncogenesis and likely explains why genetic or epigenetic loss of the *CDKN2A* locus is a particularly common event in tumors driven through this KRAS-BRAF-MAPK axis (Figure 4B). Interestingly, this induction of *CDKN2A* also occurs with oncogenes that intrinsically bypass the requirement for CDK4/6 [80,81]. For example, tumors driven by *RB* loss or *CCNE1* amplification almost invariably overexpress p16INK4A [56,82,83]. This is likely an epigenetic vestige of the oncogenic stress mediated by loss of RB or Cyclin E gain; however, in this context, cell division can continue with high p16INK4A (Figure 4B).

## 5. Therapy Resistance with Targeted Therapy

Most therapies that were developed to target oncogenic or mitogenic signaling (e.g., EGFR, KRAS, and MEK inhibitors; endocrine therapy) have their predominant effect by arresting cells in a G1 or G0-like state [43,75,84–86]. This is because, in effect, these agents are cutting off the supply of mitogens. In a variety of contexts, this is associated with subsequent death of tumor cells as the oncogenic signal is required for viability as well as proliferation. However, particularly in advanced solid tumors, residual arrested tumor cells survive. The response of these cells is dependent upon the disease paradigm and, thus, is highly varied, including responses such as therapy-induced quiescent/dormant/senescent cells, disease-tolerant persisters, minimal residual disease cells, *etc*. [87–90]. Irrespective of the terminology used to describe them, these cells are functionally arrested with a 2N DNA content, with classical features of cell cycle inhibition (e.g., dephosphorylation of RB) but serve as a reservoir from which acquired resistance can emerge. Invariably the re-emergence of disease or progression of stable disease involves the ability of sub-clones of the original tumor population to re-enter the cell cycle [87].

Cell cycle reactivation is highly dependent on the tumor type and therapy used. With many targeted therapies, the predominant mechanism driving acquired resistance is at the level of the target and bypass signaling [91]. For example, *ESR1* and *AR* mutations and aberrations are selected during treatment of HR+/HER2− breast and prostate cancer with endocrine therapy [92–94]. Similarly, mutations of *EGFR* and *RAS* are relatively common means to bypass drug effects [95–97], spurring the development of multiple “next-generation” inhibitors [98]. Alternatively, signaling pathways can frequently restore mitogenic signaling to bypass selective inhibitors. While not as common, deregulation of the cell cycle through amplification of CDK/Cyclins or loss of RB1 have been observed following the development of resistance to targeted therapies. For example, a spectrum of non-small cell lung cancer and pancreatic ductal adenocarcinoma tumors harbor CDK or Cyclin amplifications associated with resistance to EGFR and KRAS inhibitors [99,100]. Conversely, RB deficiency is associated with resistance to endocrine therapy and targeted therapies in several paradigms. Specifically in the context of prostate cancer and non-small cell lung cancer, RB1 loss is associated with a lineage transition toward a neuroendocrine phenotype that not only deregulates the cell cycle but also diminishes the importance of EGFR or AR in the biology of these tumors [44,101–103]. This does not appear to be the case in other models (e.g., triple-negative breast cancer or bladder cancer), where RB deficiency is not linked to a neuroendocrine phenotype. Since so many mechanisms of resistance act upstream of cell cycle, there has been significant energy into directly inhibiting CDK activity.

## 6. Pharmacological CDK Inhibitors

Based on the mechanisms through which resistance to targeted therapies emerges, therapeutically targeting downstream nodes may be particularly effective. Selective CDK4/6 inhibitors were first reported over 20 years ago [104,105]. These agents exhibited efficacy in mantle cell lymphoma [106], which is driven by Cyclin D1. However, the major success of these drugs since has been in the context of HR+/HER2− breast cancer in combination with endocrine therapy [107–109]. Multiple clinical trials demonstrated substantial improvement in progression-free survival [110–112], and now CDK4/6 inhibitors are widely deployed in the treatment of breast cancer. The clinical success of these drugs in HR+/HER2− breast cancer has driven significant clinical testing. A review of clinicaltrials.gov using the search terms “palbociclib,” “ribociclib,” and “abemaciclib” returned 800 results that span most disease sites. Several recent reviews have tabulated these clinical trials with differing combination therapies [113–116]. Despite this vast amount of clinical research, the only FDA approvals for CDK4/6 inhibitors are in HR+/HER2− breast cancer. Why this is the case remains only partially understood.

While single agent CDK4/6 inhibitors at relatively high doses (*e.g*., >1 μM) can have a profound effect on many cell types, this dose is not consistent with clinical drug exposure levels. Even in preclinical models, many cells can adapt to treatment with CDK4/6 inhibitor monotherapy, including HR+/HER2− breast cancer [66,117,118]. The underlying adaptations often involve parallel CDK2 activity driving the cell cycle and bypassing the necessity of CDK4/6 activity [117,119]. This response can occur rapidly and limits the utility of CDK4/6 inhibitors. Genetic screens and analyses of acquired resistance have further illustrated that many events can mediate resistance to CDK4/6 inhibitors, typically acting by uncoupling RB activation from drug treatment [11,46,117,119–121].

In principle, the challenges of overcoming single agent resistance can be achieved through the use of combination therapies, and many are highly effective in preclinical models [120,122]. Indeed, most of the ongoing clinical trials employ some form of combination therapy. Where studied, the mechanism of cooperation is typically through impacting adaptive resistance mechanisms and inducing what is considered a “deeper” cell cycle arrest that sometimes associates with a senescent-like phenotype [123–125]. The process is modulated at least in part by the TP53 tumor suppressor [126], which indirectly controls CDK2 activity and further supports the combination with CDK2 inhibitors as discussed in more detail below. Whether ongoing clinical studies will yield more FDA approvals for CDK4/6 inhibitors remains unknown, but it is likely that CDK4/6 inhibition will be broadly considered in combination strategies moving forward.

Due to the success and challenges of CDK4/6 inhibitors, new strategies are emerging to more effectively target the cell cycle machinery therapeutically. First, one approach is to limit the toxicity of the inhibitors in the clinic, thereby enabling higher on-target activity and emulating what is observed with higher drug doses in preclinical models. The dose-limiting toxicity for palbociclib and ribociclib is neutropenia, which is believed to be due to the key role of CDK6 in features of hematopoiesis [59,127]. Therefore, drugs have been developed that are more selective for CDK4 [128]. The agent atirmociclib can be dosed at a higher level in preclinical studies, enabling better disease control [128]. Ongoing clinical trials are evaluating the ability of this CDK4-selective inhibitor to treat patients with HR+/HER2− breast cancer including those who progressed on the standard of care CDK4/6 inhibitors. Second, a number of CDK2 inhibitors have been developed. These agents have a very interesting mechanism of action and serve as a reminder of the importance of cancer genetics when considering targeting drivers of G1/S. CDK2 inhibitors are particularly active in tumors driven by Cyclin E, but are even more effective when both Cyclin E and p16INK4A, the endogenous CDK4/6 inhibitor, are highly expressed [54–57]. This molecular landscape creates a bottleneck for G1/S progression that is controlled by CDK2. Thus, ongoing clinical trials are testing CDK2 inhibitors specifically in *CCNE1*-amplified cancers [13]. Outside of tumors with this distinct cell cycle regulatory landscape, CDK2 inhibitors generally induce a G2 phase arrest [55]. In most models studied to date, there has been substantial synergy when using CDK2 and CDK4/6 inhibitors jointly, suggesting that there are ways to pharmacologically induce molecular bottlenecks similar to what is observed in *CCNE1*-amplified tumors [54–57,129]. While much of this work has involved catalytic inhibitors, CDK2-targeting PROTACs and other mechanisms to inhibit CDK activity are emerging and are currently being tested in early phase clinical trials, which have been recently reviewed [13]. Overall, the study of CDK inhibitors for cancer therapy have reinforced the plasticity and heterogeneity of cancer cell cycles.

## 7. Conclusions

While the convenient textbook depiction of cell cycle is likely here for perpetuity, heterogeneity and plasticity of cell cycle in tissues and tumors illustrates the importance of considering malleable cell cycle regulatory landscapes. This has significance relevant to the etiology of select tumors driven by permutations in the cell cycle, as well as adaptations that can occur in the context of resistance to targeted therapies.

## Abbreviations

CDK: Cyclin-dependent kinase
HER2: Human epidermal growth factor receptor 2
HR: Hormone receptor
